# Development and validation of prediction model for incident overactive bladder: The Nagahama study

**DOI:** 10.1111/iju.14887

**Published:** 2022-04-07

**Authors:** Satoshi Funada, Yan Luo, Takashi Yoshioka, Kazuya Setoh, Yasuharu Tabara, Hiromitsu Negoro, Koji Yoshimura, Fumihiko Matsuda, Orestis Efthimiou, Osamu Ogawa, Toshi A Furukawa, Takashi Kobayashi, Shusuke Akamatsu

**Affiliations:** ^1^ Department of Urology Kyoto University Graduate School of Medicine Faculty of Medicine Kyoto Japan; ^2^ Department of Health Promotion and Human Behavior Kyoto University School of Public Health Kyoto Japan; ^3^ Center for Innovative Research for Communities and Clinical Excellence (CiRC_2_LE) Fukushima Medical University Fukushima City Fukushima Japan; ^4^ Center for Genomic Medicine Kyoto University Graduate School of Medicine Faculty of Medicine Kyoto Japan; ^5^ Graduate School of Public Health Shizuoka Graduate University of Public Health Shizuoka Japan; ^6^ Department of Urology University of Tsukuba Ibaraki Japan; ^7^ Department of Urology Shizuoka General Hospital Shizuoka Japan; ^8^ Institute of Social and Preventive Medicine University of Bern Bern Switzerland; ^9^ Department of Psychiatry University of Oxford Oxford UK

**Keywords:** clinical prediction rules, cohort studies, observational study, overactive, urinary bladder, urination disorders

## Abstract

**Objectives:**

We aimed to develop models to predict new‐onset overactive bladder in 5 years using a large prospective cohort of the general population.

**Methods:**

This is a secondary analysis of a longitudinal cohort study in Japan. The baseline characteristics were measured between 2008 and 2010, with follow‐ups every 5 years. We included subjects without overactive bladder at baseline and with follow‐up data 5 years later. Overactive bladder was assessed using the overactive bladder symptom score. Baseline characteristics (demographics, health behaviors, comorbidities, and overactive bladder symptom scores) and blood test data were included as predictors. We developed two competing prediction models for each sex based on logistic regression with penalized likelihood (LASSO). We chose the best model separately for men and women after evaluating models' performance in terms of discrimination and calibration using an internal validation via 200 bootstrap resamples and a temporal validation.

**Results:**

We analyzed 7218 participants (male: 2238, female: 4980). The median age was 60 and 55 years, and the number of new‐onset overactive bladder was 223 (10.0%) and 288 (5.8%) per 5 years in males and females, respectively. The in‐sample estimates for *C*‐statistic, calibration intercept, and slope for the best performing models were 0.77 (95% confidence interval 0.74–0.80), 0.28 and 1.15 for males, and 0.77 (95% confidence interval 0.74–0.80), 0.20 and 1.08 for females. Internal and temporal validation gave broadly similar estimates of performance, indicating low optimism.

**Conclusion:**

We developed risk prediction models for new‐onset overactive bladder among men and women with good predictive ability.

**Abbreviations & Acronyms**
AUC: area under the curve
BMI: body mass index
BNP: B‐type natriuretic peptide
CI: confidence interval
DCA: decision curve analysis
eGFR: estimated glomerular filtration rate
HbA1c: hemoglobin A1c
HRQOL: health‐related quality of life
IQR: interquartile range
OAB: overactive bladder
OABSS: overactive bladder symptom score
OSA: obstructive sleep apnea
PSA: prostate‐specific antigen
ROC: receiver operating characteristic
TRIPOD: Transparent Reporting of a Multivariable Prediction Model for Individual Prognosis or Diagnosis

## Introduction

OAB is defined as “a symptom characterized by urinary urgency, with or without urgency incontinence, usually with urinary frequency and nocturia in the absence of infection or other obvious pathology.”^1^, ^2^ This is one of the common conditions among the general population: the prevalence of OAB is estimated to be around 10% to 20% and increases with age.^3^, ^4^, ^5^ Though not a life‐threatening disorder, OAB symptoms reduce HRQOL and lead to higher healthcare costs.^6^, ^7^ All over the world, and especially in aging societies, the prevalence of OAB is expected to further increase, worsening the associated HRQOL and health care costs to worsen.

Population‐based prediction models are expected to support population health planning and policy decision‐making.^8^ With regard to OAB, some behaviors, such as healthy eating habits, keeping a healthy weight, quitting smoking, and performing pelvic floor muscle exercise are recommended to keep the bladder as healthy as possible.^9^ If a reliable predictive model is developed, high‐risk subjects would be identified, and then, we could encourage them to keep such good habits early on, which may potentially prevent incident OAB and save health care costs associated with drug therapies. Making such model accessible online could further facilitate clinical decision making by health‐care providers and potential patients together.

However, to the best of our knowledge, no such model has been reported to predict new‐onset of OAB. This may be due to the lack of sufficient data to develop a predictive model. This would require a large dataset, collected using a prospective design. We have recently reported a longitudinal analysis of voiding dysfunction using a large prospective cohort data from the general population.^10^, ^11^ These data can be used to develop appropriate models for new‐onset OAB in the general population.

In this study, we aim to develop and validate models to predict incident OAB in 5 years using a large prospective cohort from the general population in Japan. In addition, as the mechanism of OAB onset is different between male and female due to factors such as the prostate, menopause, and delivery, we a priori had decided to develop a different model for each sex. To make the model easier to use, we aimed to build a web‐based application to visualize the predicted results interactively.

## Methods

This study followed the TRIPOD statement (Fig. S1).^8^ The study protocol has been published elsewhere.^12^

### Study design and source of data

This is a secondary analysis of the Nagahama study, a prospective population‐based cohort study in Japan. This cohort project is conducted by the Kyoto University, the Nagahama City Office, and the non‐profit organization Zeroji Club, and the details of the Nagahama study are reported elsewhere.^10^, ^11^ Recruitment took place between November 28, 2008 and November 28, 2010, and the participants were followed up once every 5 years after baseline assessment. The follow‐up assessment was conducted from July 28, 2013, to February 10, 2016. The Nagahama City Office managed the personal information, and each participant was given a research ID and anonymized. The cohort study was approved by the ethics committee of the Kyoto University Graduate School of Medicine (no. G278) and by the Nagahama Municipal Review Board. Written informed consent was obtained from all participants.

### Study population

Participants were recruited from the general community residents of Nagahama city in central Japan. Inclusion criteria were as follows: age 30 to 74 years, ability to independently participate in health examinations, no difficulties in communicating in Japanese, no serious diseases or other health issues, and voluntary participation. From all participants, we excluded those who have been diagnosed with OAB at baseline, based on the definition of the OABSS.^13^

### Study outcome

The outcome was new‐onset OAB at the 5‐year follow‐up assessment. We used OABSS, a self‐report measure assessing urinary urgency during the past week (Appendix S1). The questionnaire consists of the following items: (i) daytime frequency, (ii) nighttime frequency, (iii) urgency, and (iv) urgency incontinence score. OAB was defined as a total OABSS ≥3, with an urgency score (iii) ≥2.^13^

### Candidate predictor variables

Based on the literature, expert opinions, and the permissible number of variables estimated from sample size calculation (Appendix S2), we pre‐selected predictor variables and developed two models for each sex in the protocol.^11^ A total of 21 and 25 parameters of variables were included in Model 1 and Model 2, respectively, for males, and 21 and 24 parameters were included in Model 1 and Model 2, respectively, for females. Appendix S3 shows the details of the predictors.

### Statistical analysis

Models 1 and 2 were developed separately for men and women using the logistic regression model, with penalized likelihood using the LASSO penalty to avoid “overfitting” of data and reduce the predictors.^14^ It is desirable to use further penalization methods to avoid extreme predictions. Ridge, LASSO, and elastic net regression are all valid and popular penalization approaches. We selected the LASSO approach in this study, because LASSO can reduce the number of predictors, which can make it easier for a model to be applied in clinical practice. Note that LASSO performs variable selection. To find the optimal hyperparameters (*λ*) required for LASSO, we followed a 10‐fold cross‐validation. We evaluated models' performance in both discrimination and calibration.^15^ Model discrimination, i.e., the ability to distinguish the participants at high‐risk and those at low‐risk, was evaluated using the area under the ROC curve (AUC, equivalent to *C*‐statistic). Model calibration, which measures the agreement between the predictions and the observed outcomes, was evaluated with calibration intercepts and slopes and was visualized with calibration plots. Good calibration is indicated by a calibration intercept near 0 and a calibration slope near 1.^16^ To evaluate and compare the net benefit between Models 1 and 2, DCA was performed.^17^ When evaluating the model performance with the data used to develop the model, we run the risk of optimism, closely related to overfitting.^18^ To decide between the two models while avoiding optimism, we performed an internal validation via 200 bootstrap resamples to calculate optimism‐corrected *C*‐statistics, calibration intercept, and slope. In addition to that, we also performed a temporal validation by splitting the sample into 3 sets according to the year of baseline assessment (i.e., 2008, 2009, and 2010). We used the first 2 sets (2008 and 2009) as the training set, and the 2010 set as the testing set, to evaluate discrimination and calibration. We selected the final model after comparing cross‐validation performance. If performance was deemed to be similar across the models, we adopted the simpler one. We used the “*glmnet*” package in R (version 4.1.2) for our analyses.^19^ All code used for our analysis is provided in https://github.com/SatoshiFunada/2021OAB_prediction_model. After deciding on the final model, we programmed a Shiny application in R to present the prediction results interactively.^20^ There was a minor change with respect to the study's protocol. We did not use multiple imputation to address missing data and decided to go for a complete case analysis, as the missing data was less than 5% for all variables.^21^ Otherwise, we adhered to the study protocol in data cleaning, model performance evaluation, and model validation.^12^

## Results

### Baseline characteristics

**Fig. 1:**
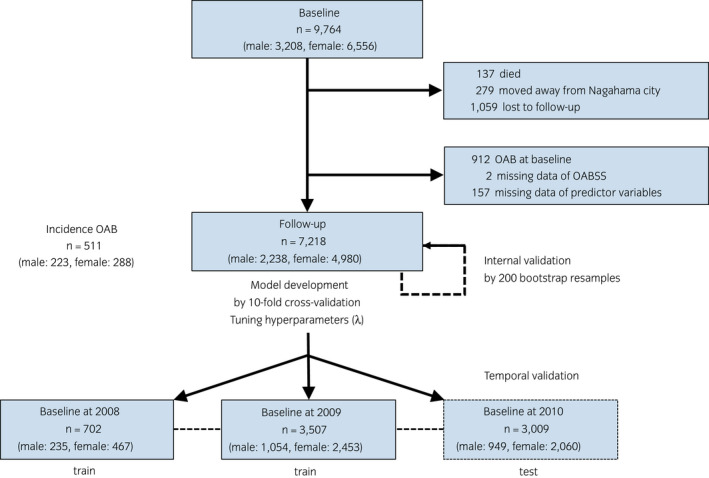
Flow diagram of participation. [Colour figure can be viewed at wileyonlinelibrary.com]

Figure 1 shows the study flow chart. From the total 9764 participants (male: 3208, female: 6556) at baseline, we excluded 1475 participants who did not attend the follow‐up assessment, and 912 participants with OAB and two with missing data for OAB at baseline (Table S1). We also excluded those with missing predictors (51 males [2.2%] and 106 females [2.1%]) and included 7218 participants (male: 2238, female: 4980) as a complete case data set. Table 1 shows the baseline characteristics excluding baseline OAB participants, and the median ages were 60 and 55 years, respectively. The number of new‐onset OAB at follow‐up assessment was 223 (10.0%) and 288 (5.8%) per 5 years in males and females, respectively (Table S2). As noted above, the data was divided into three sets according to the year of baseline assessment for a temporal validation. There were no apparent differences between the 2008 and 2009 cohort and the 2010 cohort (Table S3).

**Table 1:** Clinical characteristics of the participants excluding OAB at baseline

Baseline characteristics	Male		Female	
	Overall	Complete case	Overall	Complete case
	*N* = 2289	*N* = 2238	*N* = 5086	*N* = 4980
Year of baseline assessment				
2008	286 (12%)	235 (11%)	571 (11%)	467 (9.4%)
2009	1054 (46%)	1054 (47%)	2455 (48%)	2453 (49%)
2010	949 (41%)	949 (42%)	2060 (41%)	2060 (41%)
Age (years), median (IQR)	60 (42, 66)	60 (42, 66)	55 (40, 62)	55 (40, 62)
BMI (kg/m^2^), mean (SD)	23.5 (3.1)	23.5 (3.1)	21.6 (3.1)	21.7 (3.1)
Delivery, *n* (%)			4653 (92%)	4557 (92%)
Unknown			1	
Menopause, *n* (%)			3018 (59%)	2980 (60%)
Smoking status, *n* (%)	681 (30%)	668 (30%)	297 (5.8%)	287 (5.8%)
Alcohol habit, *n* (%)	1409 (62%)	1384 (62%)	944 (19%)	916 (18%)
Walking habit, *n* (%)	1141 (50%)	1115 (50%)	2346 (46%)	2302 (46%)
Hypertension, *n* (%)	563 (25%)	552 (25%)	807 (16%)	796 (16%)
Hyperlipidemia, *n* (%)	284 (12%)	283 (13%)	594 (12%)	589 (12%)
Diabetes, *n* (%)	196 (8.6%)	192 (8.6%)	149 (2.9%)	148 (3.0%)
Ischemic heart disease, *n* (%)	111 (4.8%)	111 (5.0%)	121 (2.4%)	119 (2.4%)
Stroke, *n* (%)	20 (0.9%)	20 (0.9%)	15 (0.3%)	15 (0.3%)
Kidney disease, *n* (%)	58 (2.6%)	55 (2.5%)	155 (3.1%)	151 (3.0%)
Unknown	17		42	
Cancer, *n* (%)	88 (3.8%)	86 (3.8%)	226 (4.4%)	225 (4.5%)
Depression, *n* (%)	71 (3.1%)	70 (3.1%)	198 (3.9%)	194 (3.9%)
Unknown	17		43	
Sleep disturbance, *n* (%)	126 (5.5%)	124 (5.5%)	437 (8.7%)	429 (8.6%)
Unknown	13		45	
OSA, *n* (%)	299 (13%)	295 (13%)	77 (1.5%)	75 (1.5%)
Prostate disease, *n* (%)	175 (7.7%)	170 (7.6%)		
Unknown	17			
Prostate cancer, *n* (%)	16 (0.7%)	16 (0.7%)		
Unknown	1			
OABSS question 1, *n* (%)				
0 score	1464 (64%)	1422 (64%)	3056 (60%)	2985 (60%)
1 score	804 (35%)	795 (36%)	1998 (39%)	1963 (39%)
2 score	21 (0.9%)	21 (0.9%)	32 (0.6%)	32 (0.6%)
OABSS question 2, *n* (%)				
0 score	889 (39%)	868 (39%)	2551 (50%)	2491 (50%)
1 score	974 (43%)	953 (43%)	1971 (39%)	1937 (39%)
2 score	319 (14%)	312 (14%)	471 (9.3%)	462 (9.3%)
3 score	107 (4.7%)	105 (4.7%)	93 (1.8%)	90 (1.8%)
OABSS question 3, *n* (%)				
0 score	1761 (77%)	1722 (77%)	4017 (79%)	3939 (79%)
1 score	520 (23%)	508 (23%)	1063 (21%)	1036 (21%)
2 score	8 (0.3%)	8 (0.4%)	6 (0.1%)	5 (0.1%)
OABSS question 4, *n* (%)				
0 score	2193 (96%)	2145 (96%)	4663 (92%)	4567 (92%)
1 score	93 (4.1%)	90 (4.0%)	395 (7.8%)	385 (7.7%)
2 score	2 (<0.1%)	2 (<0.1%)	17 (0.3%)	17 (0.3%)
3 score	1 (<0.1%)	1 (<0.1%)	9 (0.2%)	9 (0.2%)
4 score	0 (0%)	0 (0%)	2 (<0.1%)	2 (<0.1%)
HbA1c (%), mean (SD)	5.5 (0.5)	5.5 (0.5)	5.4 (0.4)	5.4 (0.4)
eGFR (mL/min/1.73 m^2^), mean (SD)	76.5 (14.7)	76.5 (14.6)	109.4 (21.5)	109.2 (21.3)
BNP (pg/mL), median (IQR)	10.2 (5.9, 17.8)	10.3 (5.9, 17.9)	13.1 (8.1, 21.4)	13.1 (8.1, 21.5)
PSA (ng/mL), median (IQR)	0.9 (0.6, 1.4)	0.9 (0.6, 1.4)		
Unknown	1			

### Model development

We did not detect problematic multicollinearity between predictor valuables by checking scatter plot matrix and calculating variance inflation factor (Table S4). Table 2 shows the covariates selected by LASSO from whole sample and the corresponding estimates of the coefficients of covariates, for all models. For both males and females, age, OABSS question 1, 2, 3, 4, HbA1c, eGFR, and BNP were selected as predictors. Smoking and diabetes were selected as a predictor for males, but both were not for females. On the other hand, BMI, alcohol habit, ischemic heart disease, sleep disturbance, and OSA were selected for females, but not for males. Prostate disease and PSA were selected for males, and delivery was selected for females.

**Table 2:** Coefficients of covariates. A missing value indicates that a covariate was not selected by the LASSO model

Coefficients	Male		Female	
	Model 1	Model 2	Model 1	Model 2
(Intercept)	−5.11	−6.17	−4.40	−6.30
Age	0.02	0.02	0.01	0.03
BMI	–	–	0.02	0.03
Delivery			−0.22	−0.41
Menopause			–	−0.36
Smoking status	0.13	0.16	–	0.01
Alcohol habit	–	–	0.10	0.22
Walking habit	–	–	–	−0.09
Hypertension	–	−0.03	–	−0.09
Hyperlipidemia	–	0.01	–	0.12
Diabetes	0.31	0.32	–	−0.08
Ischemic heart disease	–	–	0.39	0.48
Stroke	–	–	–	−0.33
Kidney disease	–	–	–	0.20
Cancer	–	–	−0.003	−0.21
Depression	–	–	–	0.09
Sleep disturbance	–	0.04	0.12	0.18
OSA	–	–	0.47	0.62
Prostate disease	0.25	0.27		
Prostate cancer	–	–		
OABSS question 1	0.21	0.22	0.31	0.37
OABSS question 2	0.35	0.34	0.46	0.48
OABSS question 3	1.12	1.13	0.88	0.94
OABSS question 4	0.08	0.11	0.83	0.84
HbA1c		0.03		0.06
eGFR		0.01		0.01
BNP		0.004		0.003
PSA		0.08		

### Model performance

Figure S2 and Table 3 show the ROC curves and the apparent *C*‐statistic, i.e., the *C*‐statistic calculated using the whole dataset for both training and testing using the LASSO models. Models 1 and 2 demonstrated similarly good discrimination for males and females, with an apparent *C*‐statistic ranging from 0.76 to 0.78 in all instances. Figure S3 and Table 3 provide calibration plots, the calibration intercept, and slope respectively. Models 1 and 2 also demonstrated similar and relatively good calibration as can be seen both visually and also judging by the value of the calibration intercept and slope for males. For females, Model 2 showed better calibration than Model 1 (intercepts were 0.20 *vs* 0.06 and slopes were 1.08 *vs* 1.03, for Model 1 *vs* 2 respectively). Figure S4 showed DCAs, and there were no apparent differences between Models 1 and 2 in both male and female.

**Table 3:** Model performance and model validation. Apparent performance was calculated using the whole dataset for both training and testing. Internal validation was via 200 bootstrap resamples. Temporal validation was by using data with year of baseline to be 2008 and 2009 to develop the model, and 2010 to test the model

	Male		Female	
	Model 1	Model 2	Model 1	Model 2
Apparent				
Discrimination				
*C*‐statistic	0.76 (0.73 to 0.79)	0.77 (0.74 to 0.80)	0.77 (0.74 to 0.80)	0.78 (0.75 to 0.80)
Calibration				
Intercept	0.29	0.28	0.20	0.06
Slope	1.15	1.15	1.08	1.03
Bootstrap				
Discrimination				
*C*‐statistic	0.75	0.75	0.75	0.76
Calibration				
Intercept	0.21	0.18	0.20	0.05
Slope	1.10	1.09	1.08	1.02
Temporal validation				
Discrimination				
*C*‐statistic	0.77 (0.73 to 0.82)	0.77 (0.73 to 0.82)	0.77 (0.73 to 0.82)	0.78 (0.73 to 0.82)
Calibration				
Intercept	1.12	0.31	0.27	0.27
Slope	1.48	1.11	1.16	1.17

### Model validation

**Fig. 2:**
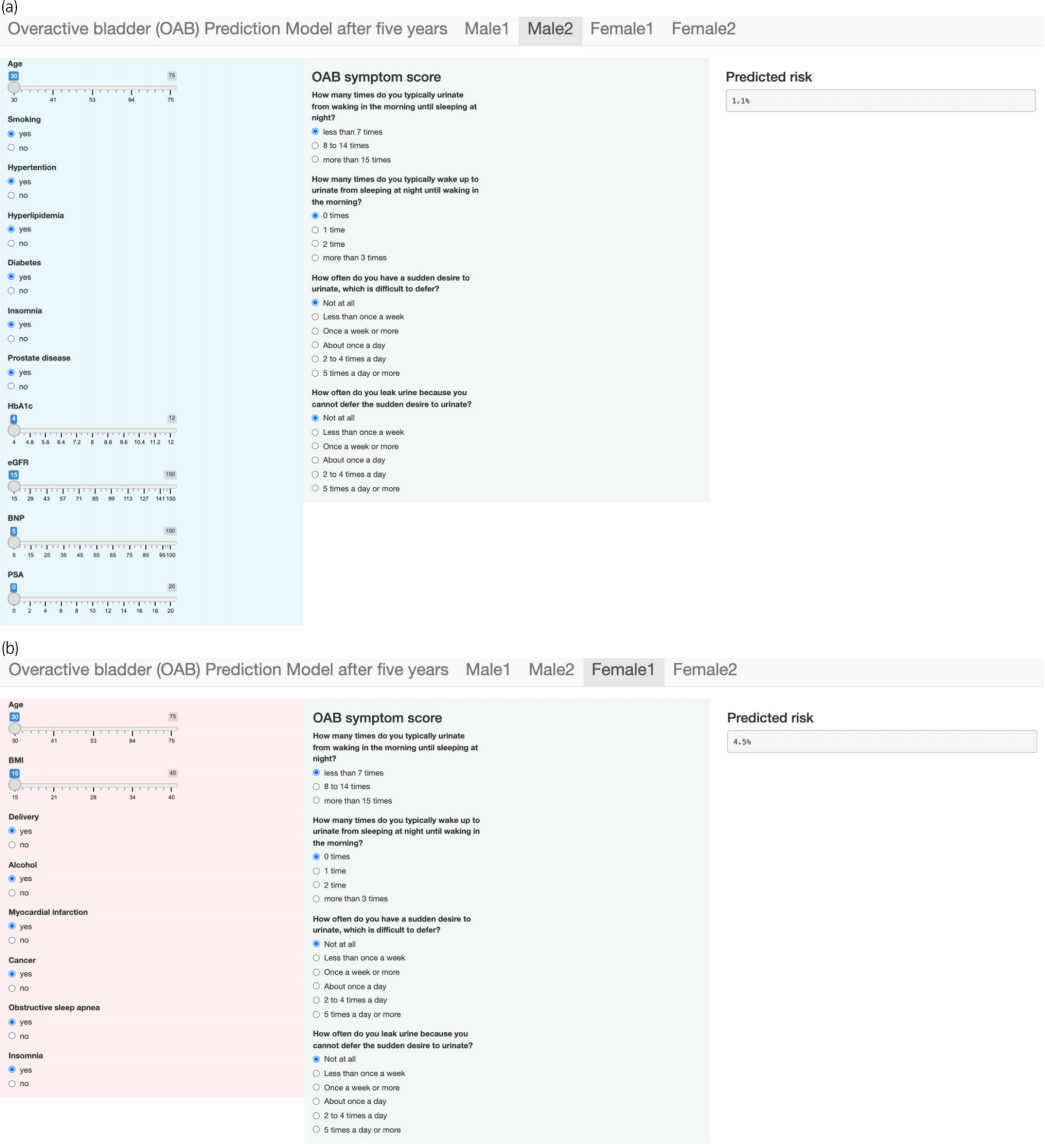
Web‐based tool to predict incident OAB in 5 years. (a) Male and (b) female. [Colour figure can be viewed at wileyonlinelibrary.com]

We performed an internal validation using 200 bootstrap resamples (Table 3). Models 1 and 2 demonstrated good discrimination, and the optimism‐corrected *C*‐statistic ranged from 0.75 to 0.76 in males and females, only slightly worse than the apparent *C*‐statistic in most cases. In males, Models 1 and 2 showed equal performance in the optimism‐corrected calibration (intercepts were 0.21 *vs* 0.18 and slopes were 1.10 *vs* 1.09, for Model 1 *vs* 2, respectively). On the other hand, Model 2 showed better calibration than Model 1 in females (the optimism‐corrected calibration intercepts were 0.20 *vs* 0.05 and slopes 1.08 *vs* 1.02, for Model 1 *vs* 2, respectively). Next, we performed a temporal validation of Models 1 and 2 in both male and female (Fig. S5; Table 3). Models 1 and 2 demonstrated good discrimination in both males and females, with a *C*‐statistic from 0.77 to 0.78. In males, Model 2 showed much better calibration than Model 1 (calibration intercepts 1.12 *vs* 0.31 and slopes 1.48 *vs* 1.11, respectively). In females, Models 1 and 2 showed similar calibration (intercept 0.27, slope 1.16 *vs* 1.17, respectively). Based on results after the internal and temporal validation, we selected Model 2 as our final model for males. For females, Model 2 performed slightly better than Model 1. However, given that differences were small, and also given that Model 1 was a simpler model, we selected Model 1 as our final model for females. We created an interactive web‐based application, in which baseline characteristics can be selected as the input, and the corresponding predicted probability of new‐onset OAB 5 years later can be generated (Fig. 2a,b and https://hxrfnn‐satoshi‐funada.shinyapps.io/OAB_prediction_model/).

## Discussion

We developed risk prediction models of new‐onset OAB for male and female in 5 years and performed internal and temporal validation using a large prospective cohort of the general population in Japan. The selected best performing prediction model for male included questionnaire assessment and blood test results as predictors, accounting for the anatomical complexity of male compared to female. On the other hand, only questionnaire assessment but no blood tests were included for female, which makes it easier to use in daily practice. Based on internally and temporally validated estimates of model performance, we deemed that both models, for men and women, had good predictive abilities.

In the model development stage, we included age, OABSS questionnaires, HbA1c, eGFR, and BNP for both males and females; however, other predictors were totally different. This is probably because the etiology of OAB is different between males and females;^4^ therefore, it was reasonable to develop prediction models separately for males and females. In terms of sex‐specific predictors, prostate disease and PSA had increased the risk of the incident OAB in male, which is consistent with previous reports.^22^, ^23^ On the other hand, our study indicated that delivery was shown to reduce incident OAB in females, which is different from previous reports.^24^, ^25^ This study was performed in a rural area, and most female participants (92%) have experienced delivery at baseline. When we compared the females with or without delivery experience, females without delivery were younger, but had a higher percentage of smokers and more comorbidities of cancer and depression than those with delivery. Therefore, females without delivery (8.5%) were a minority and may be unhealthy participants among the young general population in Nagahama cohort. This could explain why the lack of experience of delivery at baseline, in turn, increased the risk of incident OAB.

We found that for males, the prediction model including results from blood tests in the predictor list (i.e., Model 2) was better than that with only the questionnaire data. In general, among models with equal performance, the simpler the prediction model, the better they were (“Occam's razor”). Considering the ease of use, the model not including blood tests (Model 1) would perhaps be instead recommended for males. However, in a clinical setting, serum PSA is often tested to screen for prostate cancer and is useful to predict prostate volume and lower urinary symptoms in male.^26^ Given this situation in clinical practice, we consider that the current model is acceptable to be used in clinical practice for males. Assuming that blood test could not be measured, we created web‐based applications for Models 1 and 2 for both males and females (Fig. 2a,b).

Our prediction models have some implications for clinicians and policy makers. Our models can help identify high‐risk populations of incident OAB in 5 years. This may help clinicians and policy makers deliver early interventions to such people to prevent new‐onset OAB, including encouraging them to keep healthy eating habits and maintain a healthy weight, and to performing pelvic floor muscle exercise.^9^ Since there is no established prevention strategy yet, future studies are needed to investigate the benefit of potential interventions in preventing OAB among high‐risk subjects.

This study has several strengths. First, to the best of our knowledge, these are the first published prediction models for incident OAB. Second, we used a large prospective cohort data with high follow‐up rate (85%) and few missing data (2.1%) compared with previous follow‐up studies about urinary symptoms.^27^, ^28^ Third, we developed and validated prediction models according to TRIPOD guidelines and followed a study protocol. Following the prespecified analysis plan reduces the risk of selective reporting bias.^29^ Fourth, we developed risk prediction models with good predictive ability and developed a web‐based application to increase the accessibility by a wide range of people. Our models are expected to support population health‐planning and policy decision‐making regarding the prevention of OAB and hopefully prevent the incidence.

There were several limitations in our study. First, as the study participants were healthy volunteers instead of a random sample, there may be some concerns about lack of representativeness. Second, our models did not include several possible predictors, such as prostate volume, use of some drugs such as anticholinergics, frailty, neurological disorders, and pelvic organ prolapse, which could have an influence on OAB symptoms. However, it is not pragmatic to measure all these clinical/biological markers in a large epidemiological study. Moreover, had we measured them, it would not serve the purpose of prediction in the general population either: widely informative and applicable prediction models should use easily measurable characteristics. Third, we defined new‐onset OAB only according to the criteria by OABSS at follow‐up assessment without frequency‐volume chart. As OAB symptoms may be influenced by the treatment and fluctuate over time, we may have misclassified some new‐onset OAB patients during the 5 years. Information about treatment and further follow‐up study is expected to strengthen the model accuracy. Fourth, our data were not enough to evaluate possible interactions and non‐linear relationships to improve the model performance. Fifth, the participants were between the ages of 35 and 70, and our models may not be extrapolated to other age groups. Sixth, although we examined temporal validity, the Nagahama cohort is a single cohort, and we did not perform neither geographic validation in Japan nor global external validation with a fully independent external cohort outside Japan. To evaluate the general applicability of the models, future studies are needed to demonstrate the external validity of the models with other cohort data.

In conclusion, we have developed risk prediction models for new‐onset OAB in the general population with good performance. Future studies are necessary to evaluate the generalizability of the models and develop new models with better performance, possibly including some additional strong predictors. We expect that our models will help identify high‐risk populations for incident OAB, so that we could start prevention earlier.

## Author contributions

Satoshi Funada: Conceptualization; data curation; formal analysis; funding acquisition; methodology; software; visualization; writing – original draft. Yan Luo: Conceptualization; methodology; software; validation; visualization; writing – review and editing. Takashi Yoshioka: Conceptualization; methodology; writing – review and editing. Kazuya Setoh: Data curation; investigation; resources; writing – review and editing. Yasuharu Tabara: Data curation; funding acquisition; investigation; resources; supervision; writing – review and editing. Hiromitsu Negoro: Conceptualization; methodology; writing – review and editing. Koji Yoshimura: Conceptualization; methodology; writing – review and editing. Fumihiko Matsuda: Data curation; investigation; resources; writing – review and editing. Orestis Efthimiou: Conceptualization; formal analysis; methodology; software; supervision; validation; writing – review and editing. Osamu Ogawa: Conceptualization; supervision; writing – review and editing. Toshi A Furukawa: Conceptualization; methodology; supervision; writing – review and editing. Takashi Kobayashi: Conceptualization; project administration; supervision; writing – review and editing. Shusuke Akamatsu: Conceptualization; methodology; supervision; writing – review and editing.

## Conflict of interest

SF has a research grant from the Ministry of Education, Culture, Sports, Science and Technology of Japan, JSPS KAKENHI Grant Number JP 20 K18964 and a research grant from the KDDI Foundation. YL is receiving a Grant‐in‐Aid for JSPS Fellow (KAKENHI Grant Number 21 J15050). TY has research grants from the Ministry of Education, Culture, Sports, Science and Technology of Japan, JSPS KAKENHI Grant Number JP 21 K17228 for other works not related to this study. SA has a research grant from Astellas, grants from Astra Zeneca, grants from Tosoh. SA receives honoraria from Janssen, Astra Zeneca, Astellas, and Sanofi outside of the submitted work. TAF reports grants and personal fees from Mitsubishi‐Tanabe, personal fees from MSD, personal fees from Shionogi, personal fees from Sony, outside the submitted work. In addition, TAF has a patent 2018–177 688 concerning smartphone CBT apps pending, and intellectual properties for Kokoro‐app licensed to Mitsubishi‐Tanabe. OE was supported by the Swiss National Science Foundation (Ambizione grant number 180083). None of the contributing authors have any conflict of interest, including specific financial interests or relationships and affiliations relevant to the subject matter or materials discussed in the manuscript.

## Approval of the research protocol by an Institutional Reviewer Board

Approval of the research protocol by an Institutional Reviewer Board: G278.

## Informed consent

Informed consent was obtained from all participants.

## Registry and the Registration No. of the study/trial

Not applicable.

## Animal studies

Not applicable.

## Supporting information


**Figure S1.** TRIPOD checklist.
**Figure S2.** ROC curve. (a) Model 1 in male; (b) Model 2 in male; (c) Model 1 in female; and (d) Model 2 in female.
**Figure S3.** Calibration plot. (a) Model 1 in male; (b) Model 2 in male; (c) Model 1 in female; and (d) Model 2 in female.
**Figure S4.** DCA. (a) male and (b) female.
**Figure S5.** ROC curve and calibration plot in internal validity. ROC curve: (a) Model 1 in male, (b) Model 2 in male, (c) Model 1 in female, and (d) Model 2 in female. Calibration plot: (e) Model 1 in male, (f) Model 2 in male, (g) Model 1 in female, and (h) Model 2 in female.

**Table S1.** Clinical characteristics of the participants at baseline.
**Table S2.** OABSS at baseline and follow‐up.
**Table S3.** Clinical characteristics of the participants excluding OAB at baseline.
**Table S4.** VIF of predictor valuables.

**Appendix S1.** Overactive bladder symptom score.
**Appendix S2.** Sample size calculation.
**Appendix S3.** Candidate predictor variables.
