# Supplementary material for: Development and validation of prediction model for incident overactive bladder: The Nagahama study
Source: Int J Urol. 2022 Apr 7;29(7):748–56. doi: 10.1111/iju.14887 (PMC9546153; doi:10.1111/iju.14887)
Supplement: Supplementary file 1 — Figure S1. TRIPOD checklist. Figure S2. ROC curve. (a) Model 1 in male; (b) Model 2 in male; (c) Model 1 in female; and (d) Model 2 in female. Figure S3. Calibration plot. (a) Model 1 in male; (b) Model 2 in male; (c) Model 1 in female; and (d) Model 2 in female. Figure S4. DCA. (a) male and (b) female. Figure S5. ROC curve and calibration plot in internal validity. ROC curve: (a) Model 1 in male, (b) Model 2 in male, (c) Model 1 in female, and (d) Model 2 in female. Calibration plot: (e) Model 1 in male, (f) Model 2 in male, (g) Model 1 in female, and (h) Model 2 in female. [file IJU-29-748-s002.docx]

**Supplementary Fig. 1**

**Supplementary Fig. 2**

**Supplementary Fig. 3**

**Supplementary Fig. 4** ****

**Supplementary Fig. 5**
