# Supplementary material for: Development and validation of prediction model for incident overactive bladder: The Nagahama study
Source: Int J Urol. 2022 Apr 7;29(7):748–56. doi: 10.1111/iju.14887 (PMC9546153; doi:10.1111/iju.14887)
Supplement: Supplementary file 2 — Table S1. Clinical characteristics of the participants at baseline. Table S2. OABSS at baseline and follow‐up. Table S3. Clinical characteristics of the participants excluding OAB at baseline. Table S4. VIF of predictor valuables. [file IJU-29-748-s003.docx]

| Supplementary table 1. Clinical characteristics of the participants at baseline. | | | | |  | |  | | |  |  |
| --- | --- | --- | --- | --- | --- | --- | --- | --- | --- | --- | --- |
|  | Male | | | Female | | | | | | | |
|  | Overall | Loss to follow-up | OAB at baseline | Overall | | Loss to follow-up | | OAB at baseline | | |  |
| Baseline characteristics | n = 3,208 | n = 552 | n = 366 | n = 6,556 | | n = 923 | | n = 546 | | |  |
| Year of baseline assessment |  |  |  |  | |  | |  | | |  |
| 2008 | 375 (12%) | 48 (8.7%) | 41 (11%) | 737 (11%) | | 102 (11%) | | 63 (12%) |  |  |  |
| 2009 | 1,506 (47%) | 267 (48%) | 184 (50%) | 3,108 (47%) | | 385 (42%) | | 268 (49%) |  |  |  |
| 2010 | 1,327 (41%) | 237 (43%) | 141 (39%) | 2,711 (41%) | | 436 (47%) | | 215 (39%) |  |  |  |
| Age (yr), median (IQR) | 60 (42, 67) | 55 (37, 66) | 66 (61, 70) | 55 (39, 63) | | 49 (36, 66) | | 62 (53, 68) |  |  |  |
| BMI (kg/m^2^), mean (SD) | 23.4 (3.1) | 23.3 (3.2) | 23.0 (2.8) | 21.7 (3.2) | | 21.8 (3.7) | | 22.7 (3.6) |  |  |  |
| Delivery, n (%) |  |  |  | 623 (9.5%) | | 147 (16%) | | 44 (8.1%) |  |  |  |
| Menopause, n (%) |  |  |  | 3,921 (60%) | | 462 (50%) | | 440 (81%) |  |  |  |
| Smoking status, n (%) | 989 (31%) | 207 (38%) | 100 (27%) | 426 (6.5%) | | 96 (10%) | | 33 (6.0%) |  |  |  |
| Alcohol habit, n (%) | 1,996 (62%) | 325 (59%) | 261 (71%) | 1,227 (19%) | | 171 (19%) | | 111 (20%) |  |  |  |
| Walking habit, n (%) | 1,604 (50%) | 268 (49%) | 194 (53%) | 3,021 (46%) | | 421 (46%) | | 253 (46%) |  |  |  |
| Hypertension, n (%) | 811 (25%) | 108 (20%) | 139 (38%) | 1,111 (17%) | | 162 (18%) | | 142 (26%) |  |  |  |
| Hyperlipidemia, n (%) | 382 (12%) | 50 (9.1%) | 48 (13%) | 772 (12%) | | 84 (9.1%) | | 93 (17%) |  |  |  |
| Diabetes, n (%) | 308 (9.6%) | 56 (10%) | 55 (15%) | 207 (3.2%) | | 31 (3.4%) | | 27 (4.9%) |  |  |  |
| Ischemic heart disease, n (%) | 174 (5.4%) | 31 (5.6%) | 32 (8.7%) | 169 (2.6%) | | 20 (2.2%) | | 28 (5.1%) |  |  |  |
| Stroke, n (%) | 32 (1.0%) | 5 (0.9%) | 7 (1.9%) | 28 (0.4%) | | 6 (0.7%) | | 7 (1.3%) |  |  |  |
| Kidney disease, n (%) | 87 (2.7%) | 9 (1.6%) | 20 (5.5%) | 202 (3.1%) | | 24 (2.6%) | | 23 (4.3%) |  |  |  |
| Cancer, n (%) | 134 (4.2%) | 24 (4.3%) | 22 (6.0%) | 291 (4.4%) | | 38 (4.1%) | | 27 (4.9%) |  |  |  |
| Depression, n (%) | 114 (3.6%) | 25 (4.5%) | 18 (4.9%) | 285 (4.4%) | | 53 (5.8%) | | 34 (6.3%) |  |  |  |
| Sleep disturbance, n (%) | 209 (6.5%) | 43 (7.8%) | 40 (11%) | 617 (9.5%) | | 100 (11%) | | 80 (15%) |  |  |  |
| Obstructive sleep apnea, n (%) | 405 (13%) | 52 (9.4%) | 53 (14%) | 108 (1.6%) | | 11 (1.2%) | | 20 (3.7%) |  |  |  |
| Prostate disease, n (%) | 299 (9.4%) | 38 (6.9%) | 86 (24%) |  | |  | |  |  |  |  |
| Prostate cancer, n (%) | 26 (18%) | 4 (16%) | 4 (1.1%) |  | |  | |  |  |  |  |
| OABSS question 1, n (%) |  |  |  |  | |  | |  |  |  |  |
| 0 score | 1,911 (60%) | 332 (60%) | 115 (31%) | 3,732 (57%) | | 539 (58%) | | 137 (25%) |  |  |  |
| 1 score | 1,255 (39%) | 211 (38%) | 240 (66%) | 2,770 (42%) | | 375 (41%) | | 396 (73%) |  |  |  |
| 2 score | 40 (1.2%) | 8 (1.5%) | 11 (3.0%) | 53 (0.8%) | | 8 (0.9%) | | 13 (2.4%) |  |  |  |
| OABSS question 2, n (%) |  |  |  |  | |  | |  |  |  |  |
| 0 score | 1,125 (35%) | 205 (37%) | 31 (8.5%) | 3,081 (47%) | | 414 (45%) | | 116 (21%) |  |  |  |
| 1 score | 1,303 (41%) | 194 (35%) | 134 (37%) | 2,565 (39%) | | 362 (39%) | | 231 (42%) |  |  |  |
| 2 score | 547 (17%) | 104 (19%) | 124 (34%) | 721 (11%) | | 108 (12%) | | 142 (26%) |  |  |  |
| 3 score | 232 (7.2%) | 48 (8.7%) | 77 (21%) | 188 (2.9%) | | 38 (4.1%) | | 57 (10%) |  |  |  |
| OABSS question 3, n (%) |  |  |  |  | |  | |  |  |  |  |
| 0 score | 2,137 (67%) | 376 (68%) |  | 4,667 (71%) | | 650 (70%) | |  |  |  |  |
| 1 score | 614 (19%) | 94 (17%) |  | 1,228 (19%) | | 165 (18%) | |  |  |  |  |
| 2 score | 220 (6.9%) | 32 (5.8%) | 180 (49%) | 308 (4.7%) | | 49 (5.3%) | | 253 (46%) |  |  |  |
| 3 score | 146 (4.6%) | 30 (5.4%) | 116 (32%) | 225 (3.4%) | | 36 (3.9%) | | 189 (35%) |  |  |  |
| 4 score | 71 (2.2%) | 15 (2.7%) | 56 (15%) | 99 (1.5%) | | 16 (1.7%) | | 83 (15%) |  |  |  |
| 5 score | 18 (0.6%) | 4 (0.7%) | 14 (3.8%) | 28 (0.4%) | | 7 (0.8%) | | 21 (3.8%) |  |  |  |
| OABSS question 4, n (%) |  |  |  |  | |  | |  |  |  |  |
| 0 score | 2,906 (91%) | 498 (90%) | 215 (59%) | 5,596 (85%) | | 777 (84%) | | 156 (29%) |  |  |  |
| 1 score | 210 (6.6%) | 35 (6.4%) | 82 (22%) | 639 (9.7%) | | 101 (11%) | | 143 (26%) |  |  |  |
| 2 score | 55 (1.7%) | 11 (2.0%) | 42 (11%) | 172 (2.6%) | | 20 (2.2%) | | 135 (25%) |  |  |  |
| 3 score | 21 (0.7%) | 5 (0.9%) | 15 (4.1%) | 84 (1.3%) | | 13 (1.4%) | | 62 (11%) |  |  |  |
| 4 score | 12 (0.4%) | 1 (0.2%) | 11 (3.0%) | 56 (0.9%) | | 12 (1.3%) | | 42 (7.7%) |  |  |  |
| 5 score | 2 (<0.1%) | 1 (0.2%) | 1 (0.3%) | 8 (0.1%) | |  | | 8 (1.5%) |  |  |  |
| HbA1c (%), mean (SD) | 5.5 (0.7) | 5.6 (0.9) | 5.6 (1.0) | 5.4 (0.5) | | 5.4 (0.6) | | 5.5 (0.5) |  |  |  |
| eGFR (ml/min/1.73m^2^), mean (SD) | 76.5 (15.0) | 79.5 (16.3) | 71.8 (13.7) | 109.3 (21.7) | | 111.7 (23.1) | | 104.9 (20.9) |  |  |  |
| BNP (pg/mL), median (IQR) | 10.6 (6.0, 18.8) | 9.4 (5.4, 18.3) | 15.6 (8.2, 25.3) | 13.3 (8.2, 21.8) | | 13.2 (8.2, 21.6) | | 15.5 (9.6, 25.7) |  |  |  |
| PSA (ng/mL), median (IQR) | 0.9 (0.6, 1.4) | 0.9 (0.6, 1.4) | 1.0 (0.7, 2.0) |  | |  | |  |  |  |  |

BMI, Body mass index; BNP, B-type natriuretic peptide; eGFR, estimated glomerular filtration rate; HbA1c, hemoglobin A1c; IQR, interquartile range; OAB, overactive bladder; OABSS, overactive bladder symptom score; PSA, prostate specific antigen

| Supplementary Table 2. Overactive bladder symptom score (OABSS) at baseline and follow-up. | | | | |
| --- | --- | --- | --- | --- |
|  | Male | | Female | |
|  | N = 2,238 | | N = 4,980 | |
|  | Baseline | Follow-up | Baseline | Follow-up |
| OABSS question 1, n (%) |  |  |  |  |
| 0 score | 1,422 (64%) | 1,296 (58%) | 2,985 (60%) | 2,753 (55%) |
| 1 score | 795 (36%) | 902 (40%) | 1,963 (39%) | 2,187 (44%) |
| 2 score | 21 (0.9%) | 40 (1.8%) | 32 (0.6%) | 40 (0.8%) |
| OABSS question 2, n (%) |  |  |  |  |
| 0 score | 868 (39%) | 716 (32%) | 2,491 (50%) | 2,174 (44%) |
| 1 score | 953 (43%) | 824 (37%) | 1,937 (39%) | 1,893 (38%) |
| 2 score | 312 (14%) | 457 (20%) | 462 (9.3%) | 684 (14%) |
| 3 score | 105 (4.7%) | 241 (11%) | 90 (1.8%) | 229 (4.6%) |
| OABSS question 3, n (%) |  |  |  |  |
| 0 score | 1,722 (77%) | 1,667 (74%) | 3,939 (79%) | 4,032 (81%) |
| 1 score | 508 (23%) | 342 (15%) | 1,036 (21%) | 652 (13%) |
| 2 score | 8 (0.4%) | 112 (5.0%) | 5 (0.1%) | 154 (3.1%) |
| 3 score | 0 (0%) | 82 (3.7%) | 0 (0%) | 99 (2.0%) |
| 4 score | 0 (0%) | 28 (1.3%) | 0 (0%) | 34 (0.7%) |
| 5 score | 0 (0%) | 7 (0.3%) | 0 (0%) | 9 (0.2%) |
| OABSS question 4, n (%) |  |  |  |  |
| 0 score | 2,145 (96%) | 2,093 (94%) | 4,567 (92%) | 4,522 (91%) |
| 1 score | 90 (4.0%) | 112 (5.0%) | 385 (7.7%) | 331 (6.6%) |
| 2 score | 2 (<0.1%) | 16 (0.7%) | 17 (0.3%) | 67 (1.3%) |
| 3 score | 1 (<0.1%) | 9 (0.4%) | 9 (0.2%) | 49 (1.0%) |
| 4 score | 0 (0%) | 4 (0.2%) | 2 (<0.1%) | 11 (0.2%) |
| 5 score | 0 (0%) | 4 (0.2%) | 0 (0%) | 0 (0%) |
| New-onset OAB |  | 223 (10.0%) |  | 288 (5.8%) |

OABSS, overactive bladder symptom score

| Supplementary table 3. Clinical characteristics of the participants excluding OAB at baseline. | | | | |
| --- | --- | --- | --- | --- |
|  | Male | | Female | |
|  | 2008 to 2009 | 2010 | 2008 to 2009 | 2010 |
| Baseline characteristics | N = 1,289 | N = 949 | N = 2,920 | N = 2,060 |
| Age (yr), median (IQR) | 59 (40, 66) | 61 (46, 67) | 53 (38, 62) | 56 (44, 63) |
| BMI (kg/m^2^), mean (SD) | 23.6 (3.1) | 23.4 (3.0) | 21.6 (3.0) | 21.8 (3.2) |
| Delivery frequency, n (%) |  |  | 2,670 (91%) | 1,887 (92%) |
| Menopause, n (%) |  |  | 1,645 (56%) | 1,335 (65%) |
| Smoking status, n (%) | 395 (31%) | 273 (29%) | 184 (6.3%) | 103 (5.0%) |
| Alcohol habit, n (%) | 786 (61%) | 598 (63%) | 535 (18%) | 381 (18%) |
| Walking habit, n (%) | 620 (48%) | 495 (52%) | 1,349 (46%) | 953 (46%) |
| Hypertension, n (%) | 299 (23%) | 253 (27%) | 418 (14%) | 378 (18%) |
| Hyperlipidemia, n (%) | 159 (12%) | 124 (13%) | 314 (11%) | 275 (13%) |
| Diabetes, n (%) | 101 (7.8%) | 91 (9.6%) | 84 (2.9%) | 64 (3.1%) |
| Ischemic heart disease, n (%) | 67 (5.2%) | 44 (4.6%) | 75 (2.6%) | 44 (2.1%) |
| Stroke, n (%) | 12 (0.9%) | 8 (0.8%) | 9 (0.3%) | 6 (0.3%) |
| Kidney disease, n (%) | 37 (2.9%) | 18 (1.9%) | 93 (3.2%) | 58 (2.8%) |
| Cancer, n (%) | 40 (3.1%) | 46 (4.8%) | 137 (4.7%) | 88 (4.3%) |
| Depression, n (%) | 41 (3.2%) | 29 (3.1%) | 125 (4.3%) | 69 (3.3%) |
| Sleep disturbance, n (%) | 75 (5.8%) | 49 (5.2%) | 258 (8.8%) | 171 (8.3%) |
| Obstructive sleep apnea, n (%) | 166 (13%) | 129 (14%) | 43 (1.5%) | 32 (1.6%) |
| Prostate disease, n (%) | 91 (7.1%) | 79 (8.3%) |  |  |
| Prostate cancer, n (%) | 7 (0.5%) | 9 (0.9%) |  |  |
| OABSS question 1, n (%) |  |  |  |  |
| 0 score | 797 (62%) | 625 (66%) | 1,757 (60%) | 1,228 (60%) |
| 1 score | 477 (37%) | 318 (34%) | 1,145 (39%) | 818 (40%) |
| 2 score | 15 (1.2%) | 6 (0.6%) | 18 (0.6%) | 14 (0.7%) |
| OABSS question 2, n (%) |  |  |  |  |
| 0 score | 500 (39%) | 368 (39%) | 1,493 (51%) | 998 (48%) |
| 1 score | 541 (42%) | 412 (43%) | 1,109 (38%) | 828 (40%) |
| 2 score | 186 (14%) | 126 (13%) | 268 (9.2%) | 194 (9.4%) |
| 3 score | 62 (4.8%) | 43 (4.5%) | 50 (1.7%) | 40 (1.9%) |
| OABSS question 3, n (%) |  |  |  |  |
| 0 score | 978 (76%) | 744 (78%) | 2,294 (79%) | 1,645 (80%) |
| 1 score | 306 (24%) | 202 (21%) | 624 (21%) | 412 (20%) |
| 2 score | 5 (0.4%) | 3 (0.3%) | 2 (<0.1%) | 3 (0.1%) |
| OABSS question 4, n (%) |  |  |  |  |
| 0 score | 1,243 (96%) | 902 (95%) | 2,686 (92%) | 1,881 (91%) |
| 1 score | 45 (3.5%) | 45 (4.7%) | 216 (7.4%) | 169 (8.2%) |
| 2 score |  |  | 13 (0.4%) | 4 (0.2%) |
| 3 score | 1 (<0.1%) | 2 (0.2%) | 3 (0.1%) | 6 (0.3%) |
| 4 score |  |  | 2 (<0.1%) | 0 (0%) |
| HbA1c (%), mean (SD) | 5.5 (0.6) | 5.5 (0.5) | 5.4 (0.4) | 5.5 (0.4) |
| eGFR (ml/min/1.73m^2^), mean (SD) | 77.0 (14.8) | 75.8 (14.2) | 110.7 (22.1) | 107.0 (20.0) |
| BNP (pg/mL), median (IQR) | 9.8 (5.8, 16.8) | 10.9 (6.2, 19.4) | 12.9 (8.1, 21.0) | 13.4 (8.1, 22.0) |
| PSA (ng/mL), median (IQR) | 0.8 (0.6, 1.3) | 0.9 (0.6, 1.4) |  |  |
|  |  |  |  |  |
| New-onset OAB | 122 (9.5%) | 101 (11%) | 174 (6.0%) | 114 (5.5%) |

BMI, Body mass index; BNP, B-type natriuretic peptide; eGFR, estimated glomerular filtration rate; HbA1c, hemoglobin A1c; IQR, interquartile range; OAB, overactive bladder; OABSS, overactive bladder symptom score; PSA, prostate specific antigen

| Supplementary table 4. Variance inflation factor (VIF) of predictor valuables. | | | | |
| --- | --- | --- | --- | --- |
|  | Male | | Female | |
| Predictor valuables | Model 1 | Model 2 | Model 1 | Model 2 |
| Age | 1.39 | 1.69 | 3.69 | 4.13 |
| BMI | 1.10 | 1.17 | 1.12 | 1.16 |
| Delivery |  |  | 1.09 | 1.09 |
| Menopause |  |  | 3.28 | 3.32 |
| Smoking status | 1.11 | 1.13 | 1.06 | 1.06 |
| Alcohol habit | 1.03 | 1.04 | 1.03 | 1.03 |
| Walking habit | 1.05 | 1.05 | 1.09 | 1.09 |
| Hypertension | 1.22 | 1.23 | 1.23 | 1.24 |
| Hyperlipidemia | 1.10 | 1.11 | 1.08 | 1.09 |
| Diabetes | 1.08 | 1.56 | 1.04 | 1.30 |
| Ischemic heart disease | 1.04 | 1.06 | 1.08 | 1.08 |
| Stroke | 1.04 | 1.06 | 1.03 | 1.03 |
| Kidney disease | 1.03 | 1.04 | 1.01 | 1.03 |
| Cancer | 1.21 | 1.21 | 1.04 | 1.04 |
| Depression | 1.14 | 1.14 | 1.12 | 1.12 |
| Sleep disturbance | 1.15 | 1.15 | 1.19 | 1.19 |
| Obstructive sleep apnea | 1.04 | 1.04 | 1.02 | 1.02 |
| Prostate disease | 1.14 | 1.16 |  |  |
| Prostate cancer | 1.25 | 1.25 |  |  |
| OABSS question 1 | 1.07 | 1.08 | 1.06 | 1.06 |
| OABSS question 2 | 1.29 | 1.30 | 1.20 | 1.20 |
| OABSS question 3 | 1.12 | 1.12 | 1.24 | 1.24 |
| OABSS question 4 | 1.15 | 1.15 | 1.28 | 1.28 |
| HbA1c |  | 1.69 |  | 1.42 |
| eGFR |  | 1.31 |  | 1.34 |
| BNP |  | 1.14 |  | 1.14 |
| PSA |  | 1.08 |  |  |

BMI, Body mass index; BNP, B-type natriuretic peptide; eGFR, estimated glomerular filtration rate; HbA1c, hemoglobin A1c; OABSS, overactive bladder symptom score; PSA, prostate specific antigen
