# Supplementary material for: Development and validation of prediction model for incident overactive bladder: The Nagahama study
Source: Int J Urol. 2022 Apr 7;29(7):748–56. doi: 10.1111/iju.14887 (PMC9546153; doi:10.1111/iju.14887)
Supplement: Supplementary file 3 — Appendix S1. Overactive bladder symptom score. Appendix S2. Sample size calculation. Appendix S3. Candidate predictor variables. [file IJU-29-748-s001.docx]

**Supplementary Appendix 1**

***Overactive bladder symptom score (OABSS)***

The overactive bladder symptom score (OABSS) has been validated by Homma et al.^1^ OABSS is a self-report 4-item questionnaire assessing urinary urgency during the last week. The questionnaire consists of the following items: (i) ﻿How many times do you typically urinate from waking in the morning until sleeping at night? (ii) ﻿How many times do you typically wake up to urinate from sleeping at night until waking in the morning? (iii) ﻿How often do you have a sudden desire to urinate, which is difficult to defer? (iv) ﻿How often do you leak urine because you cannot defer the sudden desire to urinate? Daytime frequency score (i) is scored on a 3-point scale (range, 0 to 2), nighttime frequency score on a 4-point scale (range, 0 to 3) and urgency (iii) and urgency incontinence (iv) on 6-point scale (range, 0 to 5). The total score ranges from 0 to 15. OAB is defined as a total OABSS score ≥3, with an urgency score (iii) ≥2.

**Supplementary Appendix 2**

***Sample size calculation***

We performed sample size calculations to build a prediction model using the criteria recommended by Riley et al^2^ and used R package, “*pmsampsize*”.^3^ As there had been no prediction model of incident OAB previously, we had set R^2^ = 0.10 as a conservative choice in this study. Based on the number of complete cases (male: 2,238, female: 4,980) and events in our dataset (male: 223, female: 288) and the selected value for R^2^, we calculated the upper limit of the number of predictors to be 26 for the model for males and 58 for females.

**Supplementary Appendix 3**

***Candidate predictor variables***

Based on previous reports^4–9^ and expert opinion, we included the following candidate predictors variables which were measured at baseline; demographic variables (age, body mass index [BMI], history of delivery and menopause), health behaviors (smoking status, alcohol habit and walking habit) and comorbidities (hypertension, hyperlipidemia, diabetes, ischemic heart disease, stroke, kidney disease, cancer, depression, sleep disturbance, obstructive sleep apnea (OSA), prostate disease and prostate cancer), questionnaires specific to OAB (OABSS question 1, question 2, question 3 and question 4) and blood test results (hemoglobin A1c [HbA1c], the estimated glomerular filtration rate [eGFR], B-type natriuretic peptide [BNP] and prostate specific antigen [PSA]). Trained physicians and research assistants administered the standardized questionnaire in which participants provided clinical background information, such as lifestyle and medical history. Anthropometric and physiological measurements were taken by trained nurses.

Age was treated as continuous value. BMI was calculated as continuous values using height and weight data. Smoking status was categorized as a dichotomous variable either current or none smoker. Alcohol habit was categorized as a dichotomous variable either current or none drinker. Walking habit was categorized as a dichotomous variable by the questionnaire: walking for ≥1 h or <1 h. Delivery was categorized as a dichotomous variable either experienced or not. History and medical comorbidities (menopause, hypertension, hyperlipidemia, diabetes, ischemic heart disease, stroke, kidney disease, cancer, depression, sleep disturbance, OSA, benign prostate disease, prostate cancer) was categorized as a dichotomous variable by the questionnaire: yes or no. OABSS question 1, question 2, question 3 and question 4 was treated as continuous variables. Blood samples (HbA1c, BNP, creatine, PSA) was used as continuous values. eGFR was calculated from serum creatinine levels using the following formula: 194 × serum creatinine^−1.094^ × age^−0.287^ (×0.739 if female). We pre-selected predictor variables and developed two models as follows;

- Model 1, including demographic variables (age, BMI, history of delivery and menopause), health behaviors (smoking status, alcohol habit and walking habit) and comorbidities (hypertension, hyperlipidemia, diabetes, ischemic heart disease, stroke, kidney disease, cancer, depression, sleep disturbance, OSA, prostate disease and prostate cancer) and OABSS question 1, question 2, question 3 and question 4.
- Model 2 consisting of the predictors of Model 1 plus blood test results HbA1c, eGFR, BNP and PSA.

A total of 21 and 25 parameters of variables were included in model 1 and model 2, respectively, for males, and 21 and 24 parameters were included in model 1 and model 2, respectively, for females.

**Reference**

1. Homma Y, Yoshida M, Seki N, et al: Symptom assessment tool for overactive bladder syndrome-overactive bladder symptom score. Urology 2006; **68**: 318–323.

2. Riley RD, Snell KI, Ensor J, et al: Minimum sample size for developing a multivariable prediction model: PART II - binary and time-to-event outcomes. Stat Med 2019; **38**: 1276–1296.

3. Ensor J, Martin EC and Riley RD: pmsampsize: Calculates the Minimum Sample Size Required for Developing a Multivariable Prediction Model. : 7.

4. Dallosso HM, McGrother CW, Matthews RJ, et al: The association of diet and other lifestyle factors with overactive bladder and stress incontinence: A longitudinal study in women. BJU International 2003; **92**: 69–77.

5. Link CL, Steers WD, Kusek JW, et al: The association of adiposity and overactive bladder appears to differ by gender: Results from the boston area community health survey. Journal of Urology 2011; **185**: 955–963.

6. Ikeda Y, Nakagawa H, Ohmori-Matsuda K, et al: Risk factors for overactive bladder in the elderly population: a community-based study with face-to-face interview. Int J Urol 2011; **18**: 212–218.

7. Ohgaki K, Horiuchi K and Kondo Y: Association between metabolic syndrome and male overactive bladder in a Japanese population based on three different sets of criteria for metabolic syndrome and the Overactive Bladder Symptom Score. Urology 2012; **79**: 1372–8.

8. Hirayama A, Torimoto K, Mastusita C, et al: Risk factors for new-onset overactive bladder in older subjects: results of the Fujiwara-kyo study. Urology 2012; **80**: 71–6.

9. Kurita N, Yamazaki S, Fukumori N, et al: Overactive bladder symptom severity is associated with falls in community-dwelling adults: LOHAS study. BMJ Open 2013; **3**.
